# Carboxylesterase converts Amplex red to resorufin: Implications for mitochondrial H_2_O_2_ release assays

**DOI:** 10.1016/j.freeradbiomed.2015.11.011

**Published:** 2016-01

**Authors:** Satomi Miwa, Achim Treumann, Amy Bell, Giulio Vistoli, Glyn Nelson, Sam Hay, Thomas von Zglinicki

**Affiliations:** a Institute for Cell & Molecular Biosciences and Newcastle University Institute for Ageing, Ageing Research Laboratories, Campus for Ageing and Vitality, Newcastle University, Newcastle upon Tyne NE4 5PL, UK; b Newcastle University Protein and Proteome Analysis, Devonshire Building, Devonshire Terrace, Newcastle upon Tyne NE1 7RU, UK; c Department of Pharmaceutical Sciences, University of Milan, via Mangiagalli, 25, I-20133 Milan, Italy; d Bioimaging Unit, Medical School, Newcastle University, Framlington Place, Newcastle upon Tyne NE2 4HH, UK; e Manchester Institute of Biotechnology and Faculty of Life Sciences, The University of Manchester, 131 Princess Street, Manchester M1 7DN, UK

**Keywords:** Amplex Red, Mitochondria, Liver, Reactive oxygen species, Measurement, Carboxylesterase

## Abstract

Amplex Red is a fluorescent probe that is widely used to detect hydrogen peroxide (H_2_O_2_) in a reaction where it is oxidised to resorufin by horseradish peroxidase (HRP) as a catalyst. This assay is highly rated amongst other similar probes thanks to its superior sensitivity and stability. However, we report here that Amplex Red is readily converted to resorufin by a carboxylesterase without requiring H_2_O_2_, horseradish peroxidase or oxygen: this reaction is seen in various tissue samples such as liver and kidney as well as in cultured cells, causing a serious distortion of H_2_O_2_ measurements. The reaction can be inhibited by Phenylmethyl sulfonyl fluoride (PMSF) at concentrations which do not disturb mitochondrial function nor the ability of the Amplex Red-HRP system to detect H_2_O_2._*In vitro* experiments and *in silico* docking simulations indicate that carboxylesterases 1 and 2 recognise Amplex Red with the same kinetics as carboxylesterase-containing mitochondria. We propose two different approaches to correct for this problem and re-evaluate the commonly performed experimental procedure for the detection of H_2_O_2_ release from isolated liver mitochondria. Our results call for a serious re-examination of previous data.

## Highlights

• The H_2_O_2_ probe Amplex Red crosses the mitochondrial membrane.
• Carboxylesterase catalyses HRP-independent oxidation of Amplex Red.
• This effect needs to be blocked to enable correct H_2_O_2_ measurements in many tissues.
• PMSF inhibits HRP-independent conversion of Amplex Red.

## Graphical abstract

**Figure:**
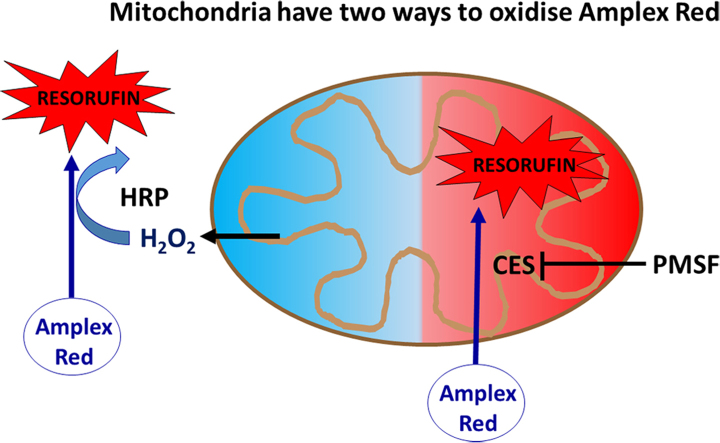

## 1 Introduction

Reactive oxygen species (ROS) are fundamentally involved in aerobic life. They are generated within biological systems and play critical roles in most of them. For instance, there is a wealth of evidence showing that, by causing damage to macromolecules, ROS can contribute to aging processes and to the pathogenesis of multiple diseases [1]. However, they also participate in cell differentiation, tissue regeneration and cellular signalling processes that can activate a multitude of stress responses, which may support survival (review, [2]). Mitochondria play a central role in cell metabolism and are a major source of ROS in cells [3], [4], and are thus involved in many different physiological and pathological processes [5], [6]. Therefore, there is a substantial need for simple but reliable and precise techniques to measure mitochondrial ROS production. Probes that change their fluorescence when oxidized provide convenient, sensitive, and versatile means for detecting ROS. Many probes with similar function have been used, such as scopoletin [7], p-hydroxyphenylacetate [8], and homovanillic acid [9]. However, Amplex Red (N-acetyl-3,7-dihydroxyphenoxazine, AR) offers greater sensitivity, lower background and better stability of the resultant fluorescent product, resorufin (7-hydroxy-3H-phenoxazin-3-one) and is thus preferred [10], [11], [12], [13], [14], [15] and has been critically evaluated [16]. AR (AH_2_) is oxidised to the highly fluorescent resorufin (A) by hydrogen peroxide (H_2_O_2_) in a 2-electron oxidation reaction catalysed by horseradish peroxidase (HRP), resulting in a 1:1 overall stoichiometry:

HRP + H_2_O_2_ → Compound I

Compound I + AH_2_ → Compound II + AH*

Compound II + AH_2_ → HRP+ AH* + 2H_2_O

AH* + AH* → A + AH_2_

Overall reaction: AH_2_ + H_2_O_2_ → A + 2H_2_O

Although H_2_O_2_- and HRP-independent oxidation of AR to resorufin has been described (*e.g.* by nitric oxide and superoxide [17]), this occurs at a considerably lower yield than HRP/H_2_O_2_-mediated oxidation. Therefore, in contrast to many other fluorescent dyes directly oxidised by various types of ROS in less specific manners [12], the AR method is generally regarded as allowing full quantification of H_2_O_2_ from the resorufin fluorescence intensity. Because AR and HRP are widely considered to be incapable of crossing biological membranes [10] (and manufacturers' information), this method is extensively used to quantify the release of H_2_O_2_ from mitochondria, and has been instrumental to gaining insights into the mechanism of mitochondrial ROS production [14]. It is also being applied to measure H_2_O_2_ release from cultured cells and tissue homogenates, as well as in various enzymatic activity assays, as many enzymatic reactions produce H_2_O_2_.

One caveat of the AR assay that has been experimentally examined is its photosensitivity (reviewed in [18]). However, so far unresolved problems have been noted when the AR method was applied to certain tissues. For example, liver mitochondria result in HRP-independent conversion of AR to resorufin at a high rate even in the absence of respiratory substrate (*i.e.* with negligible oxygen consumption). This results in the raw quantitative values from liver mitochondria being much higher than those from other tissue mitochondria in similar experimental conditions and with similar oxygen consumption rates. This phenomenon has been discussed in the community but no explanation has been put forward so far. Frequently, it has simply been ignored [15], [19], [20], [21].

Here we identify carboxylesterase (CES) as an enzyme that converts AR to resorufin without requiring either oxygen, hydrogen peroxide or a peroxidase. We show that contrary to widely held beliefs, mitochondrial membranes are permeable to AR and that AR is converted to resorufin by CES in the matrix of mitochondria from tissues with high CES expression. CES can be inhibited by Phenylmethyl sulfonyl fluoride (PMSF) at doses that do not interfere with either mitochondrial function or the kinetics of the HRP-catalysed oxidation of AR by H_2_O_2_. Therefore we propose protocols for the quantification of H_2_O_2_ by the AR method in tissues, cells and mitochondria containing CES. We argue caution in interpreting previous data using the AR methods in such samples. Based on our findings, we speculate that drug metabolism may well be an under-estimated function of mitochondria, especially in tissues such as liver and kidney.

## 2 Material and methods

### 2.1 Mice

C57Bl/6 male mice were purchased from Harlan (Blackthorn, UK). ICRFa are a substrain of C57Bl/6 kept as a long-established ageing colony at Newcastle [22]. Male mice were housed as described [23]. All work complied with the guiding principles for the care and use of laboratory animals and was licensed by the UK Home Office (PPL60/3864).

### 2.2 Mitochondria preparation and subfractionation

Mitochondria from liver, brain and skeletal muscle were isolated as described [24]. Liver mitochondria were then purified using percoll gradient [24]. For subfractionation of mitochondria, 1 mg of purified mitochondria were gently mixed with 1 ml of 10 mM Tris/HCL, pH 7.4 to obtain mitoplasts and divided into two aliquots; to one aliquot 2.7 μg proteinase K was added (to shave mitoplasts). Both aliquots were left on ice for 30 min and centrifuged at 12,000*g* for 10 min at 4 °C. To obtain inner membranes, 100 mM NaCO_3_ was added to shaved mitoplasts and left on ice for 30 min, and centrifuged at 100,000*g* for 15 min at 4 °C. Protein concentration was assessed by BioRad Dc protein assay kit with BSA as standard.

The specificity of the subfractions was confirmed by western blots using Apotosis inducing factor (AIF) as a marker for intermembrane space, NDUFA9, a subunit of the electron chain transport complex I that is localized in the inner membrane, and glutamate dehydrogenase (GDH), which resides in the mitochondrial matrix.

### 2.3 AR assay with mitochondria

H_2_O_2_ release by isolated mitochondria was measured in the assay buffer containing 115 mM KCl, 10 mM KH_2_PO_4_, 2 mM MgCl_2_, 3 mM Hepes, 1 mM EGTA, 0.2% fatty acid free BSA, pH 7.2 at 37 °C, in the presence of exogenous superoxide dismutase (75 U/ml), horseradish peroxidase (HRP) (2 U/ml) and Amplex Red (50 µM) at 37 °C. The fluorescent intensity of resorufin, the oxidised product of AR, was monitored kinetically in a plate reader (FLUOstar Omega, BMG Labtech) at excitation 544 nm and emission 590 nm. The experiments were protected from light.

For the experiments to test catalase sensitivity to resorufin, the concentration of HRP was lowered to 0.05 U/ml in order for catalase to compete for H_2_O_2_. Accordingly, the H_2_O_2_ generating system (*i.e.* the concentration of mitochondria) was also lowered so that it did not exceed the capacity of H_2_O_2_ detection at the given HRP concentration.

Typically, the basal rate (with mitochondria, no substrates) was measured for 8–10 min, then respiratory substrates (either Pyruvate+Malate 5 mM or succinate 4 mM) were added to initiate respiration (and the electron transport chain-linked H_2_O_2_ release). The fluorescent intensities of the experiments were calibrated against that obtained by the addition of a known amount of H_2_O_2_ to the experimental media in the presence of AR and HRP.

As inhibitor of HRP-independent conversion of AR to resorufin, 100 µM PMSF (2 µl of 10 mM PMSF in ethanol to the 200 µl total volume) was added to the experimental medium immediately prior to the or during the measurement. Controls received ethanol only.

In the Homovanillic acid (HVA) assay, AR was replaced with HVA (4 mM) while all the other experimental conditions remained identical to the AR assay, and the fluorescent intensity was read at excitation 310 nm and emission 430 nm.

### 2.4 AR assays with non-mitochondrial samples

To monitor the conversion of AR to resorufin by different tissue samples, various organs were washed to remove blood and homogenized in PBS, and 0.4 mg/ml samples were used in PBS containing 50 μM AR. The conversion of AR to resorufin by Ces1b enzyme was monitored in double distilled water. To create anaerobic conditions, the reaction was carried out in an anaerobic chamber (Belle Technology, UK), with oxygen concentration <5 ppm.

### 2.5 Oxygen consumption measurements

Oxygen consumption rates (OCR) by isolated mitochondria were measured in a Seahorse XF24 analyzer (Seahorse Biosciences) as described [24], with either 5 mM pyruvate and malate or 4 mM succinate. The state 3 OCR was achieved by adding 4 mM ADP, state 4 with 2 μM Oligomycin, and uncoupled rates with 4 μM FCCP. 2.5 μM Antimycin was finally added to inhibit mitochondrial respiration.

The OCR during conversion of AR to resorufin by Ces1b enzyme was monitored in a high-resolution respirometer, Oxygraph-2k (Oroboros Instruments).

### 2.6 Chemicals, enzymes and antibodies

AR was purchased from Life Technologies. All other chemicals were from Sigma. Carboxylesterase 1 isoform b human was also from Sigma (E0287); according to the manufacturer, the enzyme was formulated in 0.1 M Potassium phosphate buffer. The antibodies used in western blot experiments were as follows: NDUFA9 (Abcam, ab14713, 1:1000), AIF (Cell Signalling, #4642, 1:1000), GDH (gift from Prof. Robert Lightowlers, Newcastle University, 1:500), CES1 (ab45957, 1:1000).

### 2.7 Microscopy

3D images of purified mitochondria and mitoplasts stained with AR and mitotracker green (MTG, 100 nm) were acquired using an LSM510 confocal (Zeiss, Germany) and deconvolved using Huygens software (SVI, Netherlands). Subsequently, images were analysed using average line profiles through the central plane of the stack using ImageJ (http://imagej.nih.gov/ij).

### 2.8 LCMSMS

20 pmol AR, 20 pmol resorufin standard or 20 pmol of the AR reaction mixture with CES1b in water were analysed at a flow rate of 0.5 ml/min using a gradient from 5% B ((A) 0.1% formic acid in MS grade water, (B) 0.1% formic acid in acetonitrile) to 95% B over 2 min on a Waters Acquity UPLC BFM C18 column (1.7 µM, 2.1 mM×50 mM). The eluant was monitored using a Waters Xevo G2XS QqTOF mass spectrometer operating at a scan frequency of 10 Hz either in MS mode or in MSMS mode scanning for the precursors 214.0 and 258.0 with a collision energy ramp from 25 to 40 eV. Data were acquired in the range of m/z 50 to m/z 400 at a resolution of 22,000. Using MassLynx 4.1 extracted ion chromatograms were generated for AR (m/z 258.0766), RT (1.13 min) and for resorufin (two peaks were observed at m/z 214.0514 with RTs 0.99 min (unchanged averaged peak area over the whole time course of the experiment) and RT 0.96 min (occurring only after addition of CES1b and increasing over time to saturation)). Scans were averaged across the peaks for each molecule.

### 2.9 Docking studies

The structures of AR, resorufin and 3,7-dihydroxyphenoxazine were optimized by using the B3LYP functional and the standard 6-31G(d,p) basis set as implemented in Gamess [25]. Docking simulations involved the resolved CES1 structure in complex with naloxone methiodide, a heroin analogue (PDB Id: 1MX9) [26] as well as the already published homology model of CES2 [27]. Although the resolved structure included a homoexameric assembly, the study was focused on only one CES1 monomer, the structure of which was prepared by deleting water molecules and all crystallization additives and then underwent a preliminary optimization maintaining the backbone atoms fixed to preserve the resolved folding. After deleting the bound substrate, the so optimized protein structure was utilized by the following docking simulations by using PLANTS which calculates reliable ligand poses by ant colony optimization algorithm [28]. For both CES isozymes, the search was focused within a 10 Å radius sphere around the key serine residue (Ser221 for CES1 and Ser228 for CES2), 20 poses were generated and scored by ChemPLP function and speed was set equal to 1. The so obtained best complexes were minimized by keeping all atoms outside a 10 Å radius sphere around the bound AR fixed and the optimized complexes were finally used to calculate the parameters required by the predictive equations.

## 3 Results

### 3.1 Liver mitochondria convert AR into resorufin requiring neither respiratory chain activity nor HRP

**Fig. 1:**
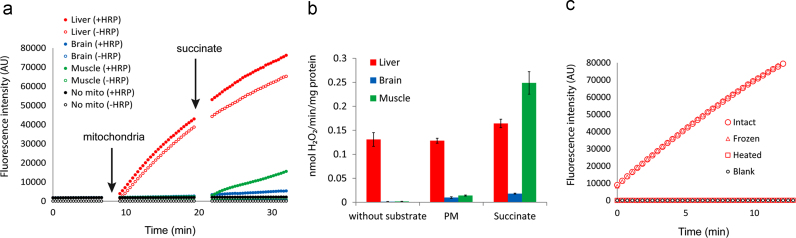
Liver mitochondria convert AR to resorufin in the absence of respiration or the catalyst HRP. (a) Traces of a representative experiment measuring conversion of AR to fluorescent resorufin by equal amounts of mitochondria (0.4 mg/ml) from mouse liver (red), muscle (green) or brain (blue) in kinetic mode (time course). Closed symbols indicate the presence of 2 U/ml HRP, while open symbols indicate no HRP in the reaction mixture. Control reactions without mitochondria are denoted by black filled circles (HRP added) or black open circles (no HRP). (b) Average conversion rates of AR to resorufin (normalized to nmol H_2_O_2_/min/mg protein) by liver, brain and muscle mitochondria (colours as above) at state 1 (without substrate) and at state 2 (with either complex I-linked substrate pyruvate and malate (PM) or complex II-linked substrate succinate) in the presence of HRP. Data are mean±SEM, 5 independent experiments. (c) AR conversion to resorufin by liver mitochondria in the absence of HRP. A representative kinetic experiment showing resorufin formation in the absence of HRP by either intact liver mitochondria (red open circles), after a freeze–thaw cycle to abolish membrane potential (red open triangles) or after heat treatment (70° for 30 min) to denature mitochondrial proteins (open squares). (For interpretation of the references to colour in this figure legend, the reader is referred to the web version of this article.)

Oxygen consumption by mitochondria at basal rate (state 1, no exogenous substrate added) is minimal and there is very little H_2_O_2_ generation. Oxygen consumption and H_2_O_2_ generation greatly increase as mitochondria are fuelled with substrate and the electron transport chain activity increases (state 2). Accordingly, the rates of AR oxidation by H_2_O_2_ to resorufin were close to zero during state 1 but significantly increased after the addition of substrate, as seen in kinetic measurements from brain or muscle mitochondria (Fig. 1a and b). In contrast, in liver mitochondria resorufin was being formed at a high rate even in the absence of respiratory substrate (state 1) (Fig. 1a and b). In brain or muscle mitochondria, resorufin formation required HRP as a catalyst, but in liver mitochondria it occurred in the absence of HRP (Fig. 1a). Furthermore, this reaction was independent of the intactness of mitochondria, because it happened even after a freeze–thaw cycle (Fig. 1c). However, heat denaturation inhibited it (Fig. 1c). The same results were obtained when fluorescence readings were made at end-point instead of employing the kinetic mode, excluding the possibility that artificial photooxidation of AR/resorufin [16], [29] was the cause of the HRP-independent oxidation of AR (data now shown).

### 3.2 PMSF inhibits the HRP-independent conversion of AR

**Fig. 2:**
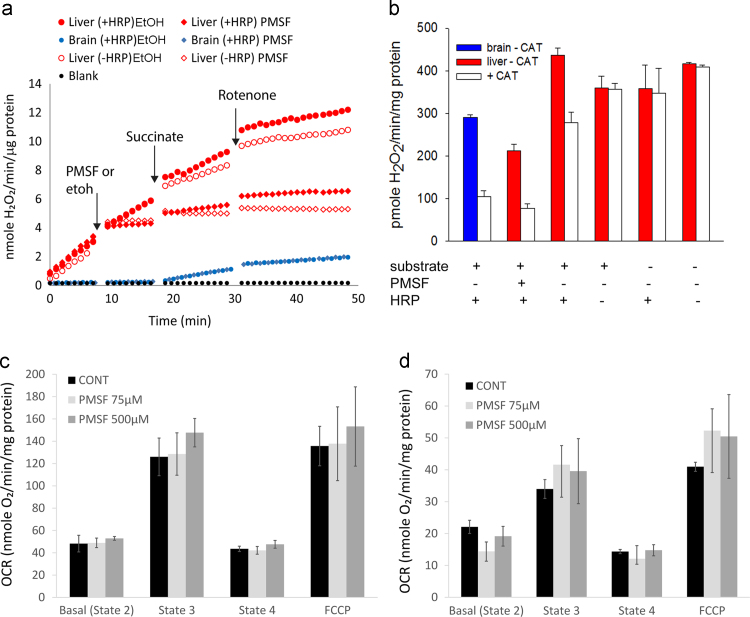
PMSF blocks HRP-independent AR conversion. (a) Representative kinetic traces from liver (red symbols) and brain (blue) mitochondria-induced AR conversion to resorufin in the presence (diamonds) or absence (circles) of 100 µM PMSF. Ethanol (EtOH) was added as a control (circles). Closed symbols indicate presence of HRP while open symbols indicate no HRP in the reaction mixture. Traces are shown in state 1 (no substrate), state 2 (succinate) and after blocking the electron flow at complex I by rotenone (2.5 µM). In all respiratory states, H_2_O_2_ release by PMSF-treated liver mitochondria is very similar to that from brain mitochondria, where PMSF does not affect the results. (b) Effects of catalase (CAT) onto AR oxidation by brain (blue) and liver (red) mitochondria. Experiments were performed without (filled bars) and with (open bars) CAT (50 U/ml) and addition of substrate (succinate), PMSF and HRP as indicated. Data are mean±SD, *n*=4. (c) PMSF does not affect oxygen consumption rates (OCR) by liver mitochondria. OCR was measured in a Seahorse XF24 analyzer with 4 mM succinate as a substrate. Mitochondria were incubated without PMSF (CONT), or with 75 µM, 500 µM PMSF. OCRs for basal, state 3 (after ADP), state 4 (after oligomycin) and uncoupled state (after FCCP) are comparable under all conditions. (d) As above, using the complex I-linked substrate, pyruvate+malate (5 mM). (For interpretation of the references to colour in this figure legend, the reader is referred to the web version of this article.)

The addition of PMSF, an inhibitor of serine proteases [30], to liver mitochondrial preparations completely inhibited the HRP-independent AR conversion to resorufin (Fig. 2a). This effect was instantaneous. Importantly, the kinetics of resorufin formation from PMSF-treated liver mitochondria showed the typical characteristics of mitochondrial H_2_O_2_ release [14], [31] as seen for instance in brain mitochondria, namely: (i) resorufin fluorescence did not increase in non-energized mitochondria, (ii) it increased with substrate addition, (iii) blocking the electron flow at complex I by rotenone decreased resorufin fluorescence when a complex II-linked substrate was used, because rotenone inhibited the reverse-electron-flow mediated superoxide production by complex I, and (iv) there was no resorufin fluorescence increase in PMSF-treated mitochondria when HRP was omitted from the reaction (Fig. 2a). Moreover, fluorescence from PMSF-treated liver mitochondria also increased following addition of a complex I-linked substrate. In this case, addition of rotenone enhanced ROS production from complex I, and accordingly the rate of fluorescence increased, albeit weakly (Supplementary Figure S1; see also Discussion). PMSF had no effects on resorufin formation from brain mitochondria under standard experimental conditions where AR was oxidised in an HRP-dependent manner by H_2_O_2_ (Fig. 2a). Adding catalase (50 U/ml) to the reaction largely inhibited the oxidation of AR by liver mitochondria in the presence of PMSF and HRP, as in energized brain mitochondria (Fig. 2b). However, the inhibitory effect of catalase was diminished in the absence of PMSF and was completely abolished in the absence of either substrate, HRP or both (Fig. 2b), indicating that the PMSF-inhibitable, HRP-independent conversion of AR by liver mitochondria was not mediated by H_2_O_2_ release. To assess whether PMSF might impact on mitochondrial function, we measured oxygen consumption rates (OCR) of liver mitochondria treated with different concentrations of PMSF in states 2, 3, 4 and after complete uncoupling using FCCP (Fig. 2c and d). Even at a PMSF concentration 5 times higher than that used for the AR experiments, OCR was unchanged under all conditions, showing that PMSF did not have adverse effects on electron transport chain activity. Together, these data suggest that PMSF inhibited the enzyme(s) responsible for the HRP-independent conversion of AR to resorufin, while leaving the capacity for HRP-dependent AR oxidation by H_2_O_2_ intact.

**Fig. 3:**
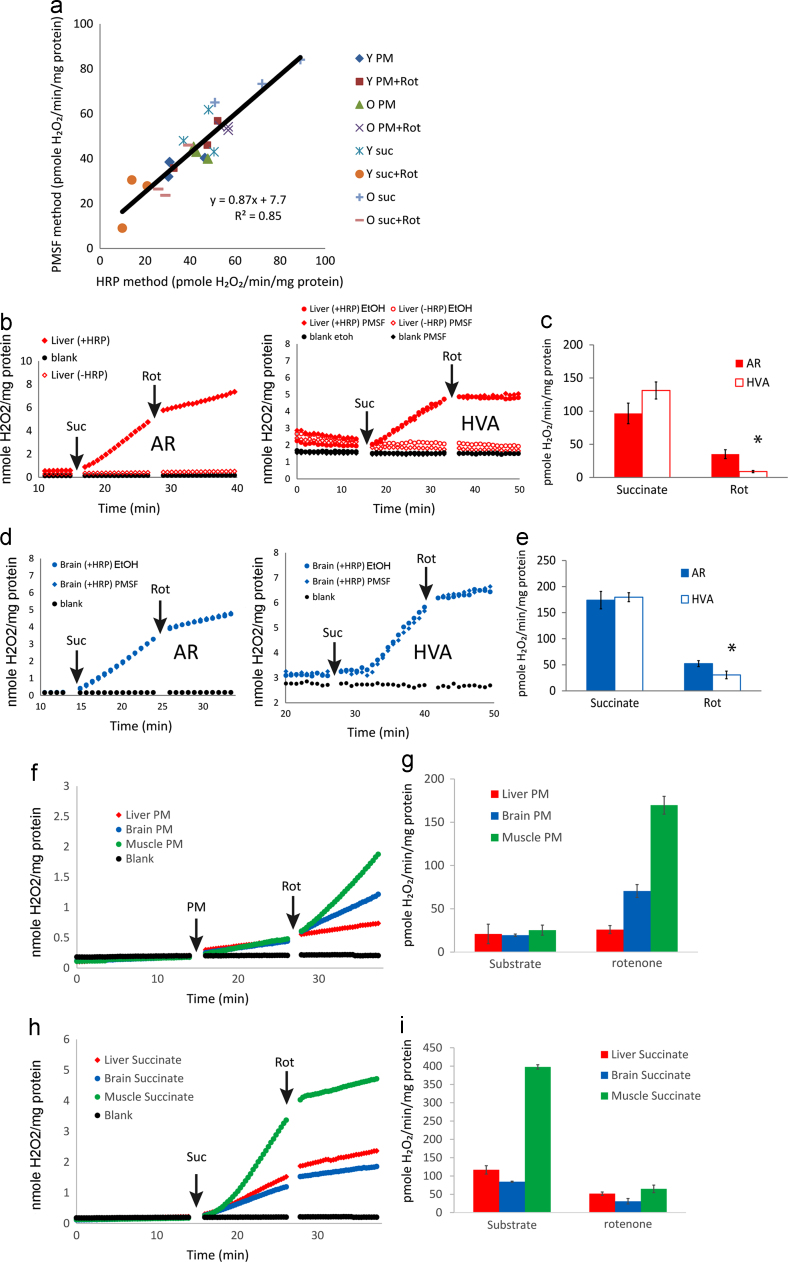
PMSF addition enables quantitative measurement of mitochondrial H_2_O_2_ release by AR. (a) H_2_O_2_ release from purified liver mitochondria measured by the HRP method and PMSF method in parallel experiments. Mitochondria were prepared from 3 young (8 months, ‘Y’) and 3 old (30 months, ‘O’) mice and the experiments were carried out in 4 conditions as follows: pyruvate+malate (PM), pyruvate+malate+rotenone (PM+Rot), succinate (suc) and succinate and rotenone (suc+Rot). The slope of the regression and the intercept are not significantly different from one or zero, respectively. (b) Representative traces from parallel experiments conducted with either AR plus PMSF (AR, left) or Homovanillic acid (HVA, right) on purified liver mitochondria with succinate (4 mM, Suc) as substrate, followed by rotenone (2.5 µM, Rot). Symbols denote the following conditions: liver mitochondria in the presence (diamonds) or absence (circles) of 100 µM PMSF. Ethanol (EtOH) was added as a control; closed symbols indicate presence of HRP in the reaction while open symbols indicate no HRP. (c) Average H_2_O_2_ release rates from succinate-energized liver mitochondria with or without rotenone measured with either AR plus PMSF (filled bars) or HVA (open bars). Data are mean±SD from 3 independent experiments. * denotes *p*<0.05. (d) Same as (b), but with brain mitochondria. (e) Same as (c), but with brain mitochondria. (f) H_2_O_2_ release rates by liver, brain and muscle mitochondria respiring complex I linked substrate (5 mM pyruvate + malate, PM) in the presence of 100 μM PMSF. Representative kinetic traces are shown in state 1 (no substrate), state 2 (after PM) and after blocking the electron flow at complex I by rotenone (2.5 µM, Rot). (g) Quantification of (f). Data are mean±SD from 3 technical repeats. (h) Same as (f), but with complex II-linked substrate (4 mM succinate, Suc). (i) Quantification of h. Data are mean±SD from 3 technical repeats.

We realized that HRP-dependent AR oxidation rates (in the absence of PMSF, calculated as the difference between the rates with and without HRP) in liver mitochondria should represent AR oxidation rates measured from PMSF treated mitochondria in the presence of HRP, that is the AR oxidation specific to mitochondrial H_2_O_2_ release: in fact they were very similar (see Fig. 2a). To test this further, we compared the results from both approaches over a range of H_2_O_2_ release rates by liver mitochondria from both young and old animals, by varying experimental conditions using complex I and II linked substrates, and with and without rotenone. Over one order of magnitude difference in H_2_O_2_ release rates, the results from both methods were in excellent agreement (Fig. 3a). Moreover, we quantitatively compared H_2_O_2_ release rates by PMSF-treated liver mitochondria as measured using AR with an alternative method, using Homovanillic acid (HVA). HVA is another commonly used HRP-dependent fluorescent probe to monitor H_2_O_2_ generation. With this probe, there was no HRP-independent HVA oxidation in liver mitochondria (Fig. 3b). H_2_O_2_ release rates with succinate as substrate determined using HVA were comparable to those of PMSF-treated mitochondria measured using AR (Fig. 3b and c). Slowing down the rate of H_2_O_2_ release by addition of rotenone was seen by both methods, but the rates measured using the HVA method were lower than those measured using the AR method (Fig. 3c). Brain mitochondria yielded similar results (Fig. 3d and e), showing a discrepancy between the two methods when H_2_O_2_ release rates are low. This is most probably due to the known lower sensitivity and thus a higher detection limit of the HVA assay [32]. In agreement with that, we could not detect H_2_O_2_ release by brain mitochondria respiring complex I linked substrate with the HVA assay unless rotenone was present, although it was clearly detectable by AR (Supplementary Figure S2). Taken together, the data indicate that in the presence of PMSF the AR methods enables for the first time realistic estimates of H_2_O_2_ release from mitochondria in different tissues including liver (Fig. 3f–i).

**Fig. 4:**
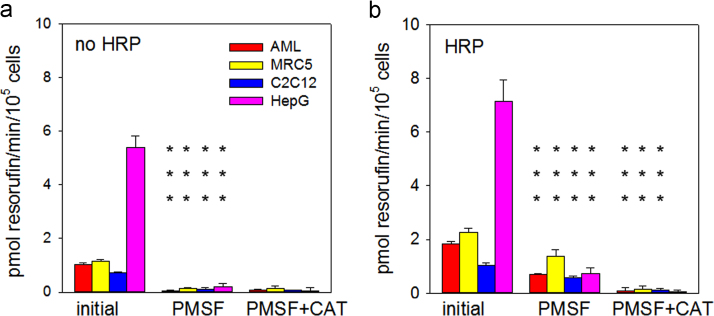
Measurement of H_2_O_2_ release from cultured cells. Cells from the indicated strains were incubated with AR in the absence (a) or presence (b) of HRP and either no further addition (initial), 100 µM PMSF, or 100 µM PMSF plus 50 U/ml catalase (CAT). Data are mean±SD from 3 experimental replicates. Asterisks indicate significant differences (***p<0.001) between PMSF and initial and between PMSF+CAT and PMSF (cell line-specific ANOVA followed by Holm–Sidak post-hoc test).

Frequently, the AR method is used to measure hydrogen peroxide release from cultured cells. We measured resorufin fluorescence generated with and without HRP, PMSF and catalase (Fig. 4a and b) from four cell lines: AML12 mouse hepatocytes; MRC5, a primary human fibroblast strain; C2C12 undifferentiated mouse myoblasts; and Hep G2 human hepatocellular carcinoma cells. All four lines showed significant HRP-independent resorufin production that was completely inhibited by PMSF (Fig. 4a). HRP-dependent, PMSF-insensitive resorufin production was sensitive to catalase (Fig. 4b), indicating that this was a correct indication of H_2_O_2_ release. It should be noted that the cell lines rank differently in terms of PMSF-dependent and –independent resorufin production rate in accordance with primary fibroblasts being more dependent on oxidative phosphorylation (and thus producing more H_2_O_2_) than immortalized and tumour cells.

### 3.3 AR crosses the mitochondrial membrane and is converted to resorufin in the matrix

**Fig. 5:**
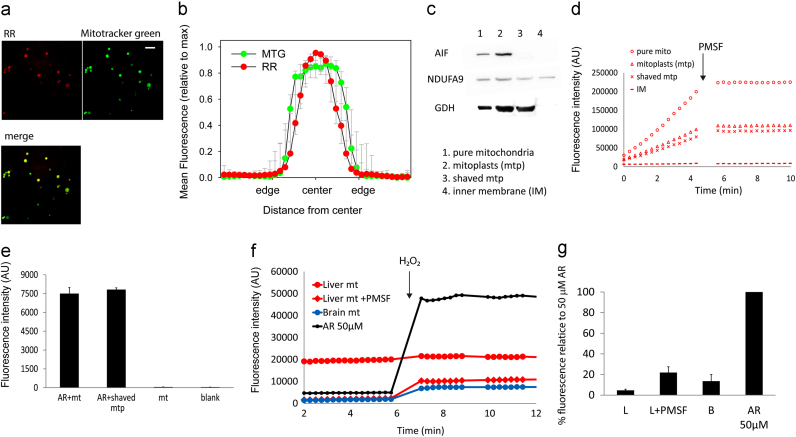
PMSF-sensitive conversion of AR to resorufin occurs in the mitochondrial matrix. (a) Confocal images of isolated liver mitochondria (average intensity projections of 10×0.1 μM confocal planes captured at 1 Airy unit) showing resorufin and Mitotracker Green fluorescence. Resorufin fluorescence can be seen throughout the mitochondria matrix. Scale bar is 5 µM. (b) Mean line profile intensities of resorufin (RR) fluorescence through the central plane image of intact mitochondria after normalising both intensity (maxima=1) and size (edges and centre defined to set all objects to an equal, arbitrary diameter). *N*=13. Full width half maximum of the resorufin distribution is smaller than that of Mitotracker Green (MTG, *p*<0.001, paired t test). (c) Western blots of mitochondrial subfraction marker proteins. AIF, apoptosis inducing factor; NDUFA9, NADH dehydrogenase 1 alpha subcomplex 9 (a component of complex I in the inner membrane); GDH, glutamate dehydrogenase. (d) Representative traces of resorufin fluorescence following incubation of the indicated liver mitochondrial subfraction with AR in the absence of HRP or substrate. All reactions are PMSF sensitive. (e) Resorufin fluorescence measured after incubation of intact liver mitochondria with AR for 10 min. Fluorescence (no PMSF, no further AR) was measured from intact mitochondria (AR+mt) and after removing the outer membrane and intermembrane space (AR+shaved mtp). Controls were mitochondria incubated without AR (mt) and blanks. Data are mean±SD, *n*=3. (f) Representative traces of resorufin fluorescence in assay medium containing AR-pre-incubated liver (red circle) or brain (blue circle) mitochondria as only source of AR. Liver mitochondria were pre-treated (red diamonds) or not (red circle) with PMSF. 50µM AR without mitochondria was used as positive control. 49 pmole H_2_O_2_ were added at the indicated time point. (g) Quantification of the experiment in **f**. Data are mean±SD, *n*=4. All values are significantly above 0 (*P*<0.05, *t*-test). (For interpretation of the references to colour in this figure legend, the reader is referred to the web version of this article.)

To identify the source of the HRP-independent AR conversion to resorufin by liver mitochondria, we first assessed the possibility of microsomal contamination. However, our mitochondrial preparations were pure; fewer than 5% of the total of the >600 detected proteins in purified liver mitochondria were of microsomal origin [24]. Moreover, confocal imaging of purified liver mitochondrial preparations showed resorufin fluorescence only in organelles which were also positive for the mitochondrial marker Mitotracker green (Fig. 5a), thus excluding non-mitochondrial organelles as sites of HRP-independent resorufin generation. Profile scanning of the confocal mitochondrial images revealed a wider signal for Mitotracker Green, while the resorufin fluorescence was focussed closer to the centre of the mitochondrial images (Fig. 5b), suggesting an intramitochondrial origin of the resorufin fluorescence.

In order to determine the submitochondrial localisation of the site of AR conversion to resorufin, mitoplasts (mitochondria without the outer membrane), ‘shaved’ mitoplasts (mitoplasts after removal of intermembrane space proteins), and inner mitochondrial membranes were prepared from purified liver mitochondria (Fig. 5c). All three fractions were tested for PMSF-sensitive AR conversion to resorufin. As seen in Fig. 5d, HRP-independent, PMSF-sensitive AR conversion to resorufin occurred in intact mitochondria and in shaved and unshaved mitoplasts, but not in the inner membrane fraction. These observations were supported by confocal imaging, showing a clear resorufin fluorescence signal also in mitoplasts (not shown). These experiments showed that the PMSF-sensitive conversion of AR did not occur on the outside of the inner mitochondrial membrane (because the inner membrane fraction was negative) or in the intra-membrane space (because rates were equal for mitoplasts and shaved mitoplasts, Fig. 5d). This suggested that AR is able to cross the inner mitochondrial membrane and that the AR-converting activity resided in the matrix. To see whether AR was also able to cross the outer membrane, we incubated liver mitochondria with AR for 10 min without PMSF, allowing for its conversion to resorufin, and divided them into two aliquots. Then the outer membrane from one aliquot of mitochondria was removed and the other served as a control. The resorufin fluorescence intensity of the mitochondria without the outer mitochondrial membrane was identical to the rest of mitochondria (Fig. 5e), confirming that the resorufin signal was not associated with the outer membrane.

To see whether AR can cross mitochondrial membranes in both directions, liver and brain mitochondria were incubated with AR for 10 min at 37 °C, washed twice and then incubated in fresh assay medium with HRP but not AR. External H_2_O_2_ (49 pmole) was then added. In this setting, mitochondria are the only source of AR. Only if the washed mitochondria retain AR and release it into the assay medium, can resorufin be generated by HRP-catalysed oxidation by H_2_O_2_. This happened for both brain and liver mitochondria (Fig. 5f), albeit the latter displayed a higher basal level of fluorescence and a lower level of resorufin formation following H_2_O_2_ addition, suggesting that a significant proportion of AR had already been converted to resorufin in liver mitochondria prior to the HRP-dependent H_2_O_2_ assay. This was confirmed if liver mitochondria were pre-treated with PMSF, leading to low basal fluorescence and more pronounced H_2_O_2_-induced resorufin formation similar to brain mitochondria (Fig. 5f). Quantification of the resultant resorufin suggested that the amount of retained AR limited H_2_O_2_ detection (Fig. 5g). Conversely, when mitochondria were pre-incubated with HRP instead of AR, and washed prior to adding AR (but not HRP) for an H_2_O_2_ assay, no conversion of AR to resorufin was observed (data not shown), confirming that AR but not HRP was able to permeate the mitochondrial membrane. Together, these data indicate that AR can transverse the mitochondrial membrane in both directions (either by diffusion or by a transporter-mediated mechanism).

### 3.4 Carboxylesterase catalyses the HRP-independent conversion of AR to resorufin

To identify enzymes that could perform the HRP-independent, PMSF-inhibitable conversion of AR to resorufin, we searched lists of proteins specific to highly purified mouse liver mitochondria [24], [33]. This suggested carboxylesterases (CESs) as possible candidates: CESs belong to the serine hydrolase family [34], [35], and PMSF targets serine residue in the active site of enzymes [30]. PMSF was reported to inhibit carboxylesterases [36], and other hydrolases [37] which have now been classified as CES. There are 20 isoforms of CES in the mouse with tissue specific distributions [38], participating in the metabolism of xenobiotics, drugs and lipids. Different CES isoforms CES1, CES3, CES5 and CES6 have been found in highly purified mouse liver mitochondria [24], [33]. The CES1 gene structure suggests a 50–60% probability for mitochondrial localisation [39].

**Fig. 6:**
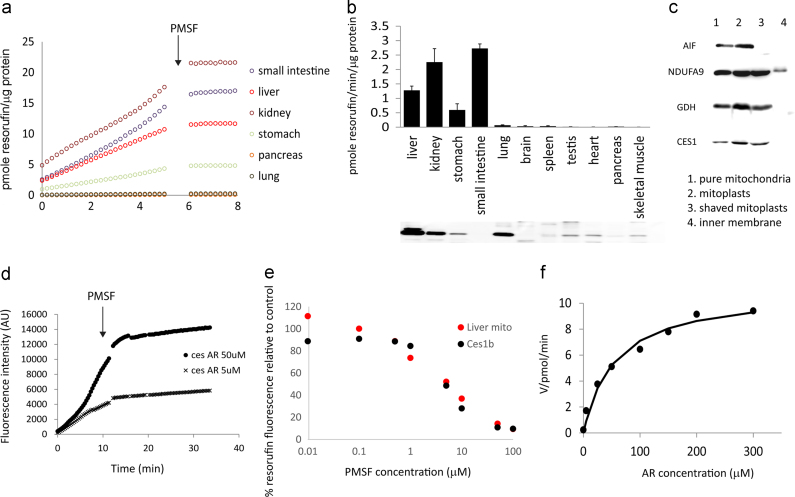
Carboxylesterase catalyses the HRP-independent conversion of AR to resorufin. (a) Representative traces of resorufin fluorescence catalyserd by the indicated tissues without addition of HRP. 100 μM PMSF was added at the indicated time point. (b) Rates of HRP-independent resorufin generation by the indicated tissues. Data are mean±SD from 2 to 4 independent experiments. In a lower panel, each tissue homogenate sample (30 µg protein) was tested for CES1 abundance by Western blot. (c) Detection of CES1 by Western blot in pure liver mitochondria (1), mitoplasts (2), shaved mitoplasts (3), but not in inner membrane subfractions (4), suggesting it is in the mitochondrial matrix. (d) Human recombinant Carboxylesterase 1 isoform b (Ces1b) converts AR to resorufin in a PMSF-sensitive manner. 100 μM PMSF was added at the indicated time point. (e) PMSF dose response curves for the AR conversion to resorufin by Ces1b (Ces1b, black circle) and liver mitochondria (Liver mito, red circle). (f) Michaelis–Menten kinetics showing the rate of resorufin production (in pmol/min) by recombinant Ces1b *vs* AR concentration. (For interpretation of the references to colour in this figure legend, the reader is referred to the web version of this article.)

Different tissues catalyse the HRP-independent conversion of AR to resorufin at different rates, however, all are inhibited by PMSF (Fig. 6a). The tissue distribution of CES1 corresponds well with the PMSF-inhibitable production of resorufin (Fig. 6b). Discrepancies in individual tissues (*e.g.*, gut and lung) might be due to cross-reactivities and/or different isoforms being expressed. Within liver mitochondria, CES1 is located in the matrix (Fig. 6c), as is the HRP-independent AR conversion to resorufin (see Fig. 5d).

Finally, human Carboxylesterase 1 isoform b (Ces1b) converts AR to resorufin in the absence of H_2_O_2_ and HRP *in vitro* (Fig. 6d). The reaction is inhibited by PMSF with an IC50 ∼5 µM, exhibiting similar behaviour to liver mitochondria (Fig. 6e). This reaction follows Michaelis–Menten kinetics, yielding a *V_max_* of 11.0±0.7 pmol/min and a *K_M_* of 55±12 µM at 12.5 U/ml, indicating that CES behaved like a typical enzyme with AR as a substrate (Fig. 6f). Following its incubation with CES for 28 h, up to 90% of the original AR molecules in the reaction were detected as resorufin (Supplementary Figure S3). The UV spectrum of the reaction product was identical to that of resorufin (not shown) and the identity of the product was confirmed by LCMSMS (Supplementary Figure S4). The reaction of AR and Ces1b was sensitive to a typical inhibitor for CES1 and CES2, bis(4-nitrophenyl)phosphate (BNPP), resulting in 80–90% inhibition at 1 µM, IC50 ∼0.15 µM similar to that of liver mitochondria (Supplementary Figure S5a). Importantly, like PMSF, BNPP at concentrations that inhibited CES efficiently did not impact on mitochondrial respiration (Supplementary Figure S5b), and conversion of AR to resorufin in the presence of BNPP required HRP (Supplementary Figure S5c). Loperamide (LPM) is known as a specific inhibitor for CES2, and CES1 has been shown to be insensitive to LPM over a range of concentrations (up to 100 µM) when classic CES substrates were used, such as 4-Nitrophenyl acetate, Fluorescein diacetate [40] or p-nitrophenyl acetate [41]. However, both the Ces1b- catalysed conversion of AR to resorufin in vitro and the conversion catalysed by liver mitochondria showed similar behaviour in the presence of LPM, with only ∼50% inhibition of activity observed in the presence of 100 µM LPM (Supplementary Figure S6). These data suggest that CES1, and possibly CES2, can account for AR conversion activity.

**Fig. 7:**
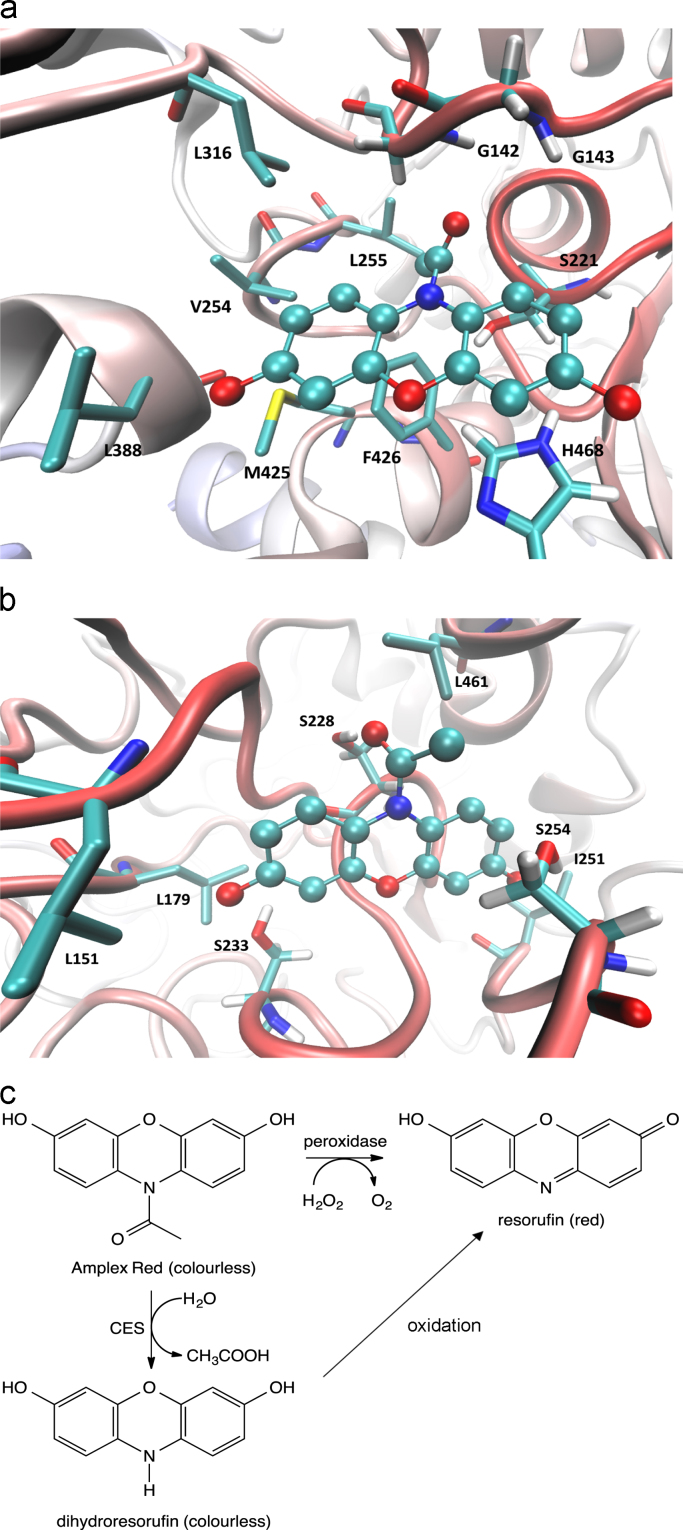
CES1 and CES2 can interact with AR. (a) Docking simulation on CES 1 and (b) on CES2 2 for AR. The substrate is shown as balls and sticks with the interacting residues in liquorice. Green colour indicates carbon, blue indicates nitrogen, red stands for oxygen and yellow for sulphur atoms. For clarity reasons, the hydrogen atoms are not displayed. (c) Proposed mechanism for CES to convert AR to resorufin. (For interpretation of the references to colour in this figure legend, the reader is referred to the web version of this article.)

To understand the potential reactions between carboxylesterases and AR, we performed docking simulations based on the resolved structure of CES1 in complex with a heroin analogue (PDB Id: 1MX9) and the published homology model of CES2 [27], [42]. Simulating the putative complex of AR within the enzymatic cavity of CES1 (Fig. 7a) showed significant complex-stabilizing interactions, revealing the labile amide moiety of AR in a position conducive to catalysis: Its carbonyl carbon atom approached the hydroxyl group of Ser221, while the carbonyl oxygen atom clearly forms H-bonds with the so-called oxyanion hole that is composed of the backbone atoms of Gly142 and Gly143. The remaining part of the substrate is substantially engaged in hydrophobic contacts. In detail, the phenoxazine ring stabilizes π–π stacking interactions with Phe101, Phe365, Phe426, and His 468, plus hydrophobic contacts involving several apolar side-chains surrounding the AR molecule (*e.g.* Val254, Leu255, Leu318, Ile359, Ile388 and Met425). Notably, Leu255 and Ala222 also contact the methyl group of the acetyl moiety. In contrast, the two hydroxyl functions were involved in not more than weak H-bonds with backbone atoms (*e.g.* the 3-hydroxyl approaches the backbone carbonyl group of His468). Together, the molecular docking simulations confirmed the overall stability of the computed complex and revealed the key role played by Van der Waals and hydrophobic interactions in both complex stabilization and undocking processes [43].

Fig. 7b shows the putative complex between AR and CES2. Again, the hydrolysable group of the substrate is placed in a position suitable for catalytic interaction since its carbonyl carbon atom conveniently approaches the key catalytic Ser228 residue. The CES2 subpocket accommodating the phenoxazine ring includes some polar residues and indeed the two hydroxyl functions are engaged in H-bonds with Ser233 and Ser254 while the phenoxazine ring elicits hydrophobic contacts involving Leu151, Leu179, Ile251, and Leu461. The comparison of the two putative complexes reveals some relevant differences mostly focused on the contacts stabilized by the phenoxazine ring. Both enzymes elicit rich networks of hydrophobic contacts which are reinforced by π–π stacking in CES1 and by H-bonds with hydroxyl functions in CES2.

Using these models together with published correlative data [42] , pKm values were predicted for the interactions of either CES1 or CES2 with AR (Supplementary Table S1), resulting in excellent agreement with the experimentally measured CES1 pKm value.

The low distances between the labile group of AR and the catalytic serine residues of either CES1 or CES2 (Supplementary Table S1) indicate AR as a possible catalytic substrate for both enzymes. Thus, we propose that CES1 and CES2 are able to convert AR to resorufin in a two-step reaction (Fig. 7c). Enzymatic data and docking simulations suggest that CES1 and CES2 act as amidases, cleaving the amide group of AR, resulting in the release of acetic acid and 3,7-dihydroxyphenoxazine (dihydroresorufin). While we cannot predict how the spontaneous oxidation of 3,7-dihydroxyphenoxazine might occur in physiological conditions, we argue that 3,7-dihydroxyphenoxazine is significantly more reactive than AR or resorufin according to its computed quantum-chemical descriptors (Supplementary Table S2). In the presence of molecular oxygen, 3,7-dihydroxyphenoxazine is readily oxidised to resorufin, forming the basis of the ‘Vanishing Valentine’ classroom experiment [44]. However, the *in vitro* conversion of AR to resorufin was observed to be as efficient in an anaerobic chamber (oxygen concentration <5 ppm) using degassed water as it was in air (Supplementary Fig. S7a), and the reaction was not inhibited by catalase (Supplementary Fig. S7b). In oxygenated solution in complete darkness, the oxygen consumption detected by high-resolution oxygraphy was very low and not different whether resorufin was produced or not (by blocking the reaction with PMSF) (Supplementary Fig. 7c–e), suggesting that the observed slope of the oxygraph trace was due to electrode drift. In any case, this slope would only equate to less than 0.0002 moles oxygen consumed per mole resorufin formed. While the mechanism of 3,7-dihydroxyphenoxazine oxidation during the CES-catalysed reaction remains unresolved, the oxidation step is unlikely to be rate-limiting.

## 4 Discussion

The two main results of our study, namely that mitochondrial membranes are permeable to AR and that carboxylesterases can convert AR to resorufin in a HRP- and oxygen-independent reaction that is inhibited by PMSF, bear a number of interesting consequences. First, they enable the sensitive and quantitative assay of H_2_O_2_ release by mitochondria from tissues, cells and mitochondria with high CES content such as liver using the AR method. We showed here that the PMSF-insensitive and the HRP-dependent part of the resorufin fluorescence were quantitative measures of H_2_O_2_. PMSF could be substituted by BNPP, as both did not affect OCR, and resorufin formation in the presence of either PMSF or BNPP was fully dependent on HRP, suggesting it was due to HRP-catalysed oxidation by H_2_O_2_.

It had previously been claimed that liver mitochondria produced more ROS than mitochondria from other tissues under comparable experimental conditions[15], [19], [20], [21] and that liver mitochondria ROS production was insensitive to variations in substrates and ETC inhibitors [45], [46], [47]. These are most probably misinterpretations due to the observed high CES activity, which masks the real H_2_O_2_-dependent signals. Our results indicated qualitatively and quantitatively similar H_2_O_2_ release rates in mitochondria from liver and other tissues (see Fig. 3f–i). Our data revealed for the first time tissue-specific differences in the characteristics of complex I-linked H_2_O_2_ release. For example, liver mitochondria are much less sensitive to rotenone in forward-electron flow H_2_O_2_ release (*i.e.* when complex I substrate was used) compared with brain or muscle mitochondria, whereas they are equally sensitive to reverse flow mediated H_2_O_2_ release (*i.e.* ∼50% reduction in the rate of H_2_O_2_ release when complex II linked substrate was used).

We verified that membranes of intact mitochondria are permeable to AR, but not to HRP. Therefore, the notion that the AR method quantitatively detects extramitochondrial release of H_2_O_2_ still holds, as long as inhibitors of HRP-independent AR conversion are added to the experiments.

Second, our data provide a rationale for a novel carboxylesterase assay by measuring the PMSF-sensitive and HRP-independent conversion of AR to resorufin. In vitro, CES converts AR quantitatively to resorufin, following strict Michaelis–Menten kinetics (Fig. 6f). In liver mitochondria *ex vivo*, this conversion of AR to resorufin changes with the physiological state of the tissue, for instance, it increases with donor age and decreases under dietary restriction (Supplementary Figure S8). However, exactly which isoforms of CES are involved in this reaction in different tissues is unknown.

Finally, our data support the speculation that mitochondria could function as sites of xenobiotic drug metabolism. If AR crosses mitochondrial membranes other molecules may have the same ability. Enzymatic reactions involving CES will frequently produce free radical species, either directly or indirectly. A localisation of carboxylesterases in the mitochondrial matrix with its high concentration of antioxidants might therefore be advantageous for the cell.

## Footnotes

Appendix A Supplementary data associated with this article can be found in the online version at doi:10.1016/j.freeradbiomed.2015.11.011.
