# Supplementary material for: Carboxylesterase converts Amplex red to resorufin: Implications for mitochondrial H2O2 release assays
Source: Free Radic Biol Med. 2016 Jan;90:173–83. doi: 10.1016/j.freeradbiomed.2015.11.011 (PMC4708625; doi:10.1016/j.freeradbiomed.2015.11.011)
Supplement: Supplementary file 1 — Supplementary material [file mmc1.docx]

**Supporting Material**

**Supporting Tables**

**Table S1: Predicted pKm values for CES1 and CES2**

| Enzyme | DistSer221/228 | MLPInS | Volume | pKm predicted | pKm exp |
| --- | --- | --- | --- | --- | --- |
| CES1 | 3.05 | -0.64 | 215.4 | 4.08 | 4.74 |
| CES2 | 2.88 | -0.36 |  | 4.66 | n.d. |

For CES1: pKm = – 1.66(± 0.151 ) MLPInS – 0.381(± 0.112) DistSer221 + 4.18(± 0.691)

For CES2: pKm = - 4.17(±0.23) MLPInS - 0.25(±0.071) DistSer228 + 2.12·10-3(±6.35·10^-4^) Volume + 3.43(±0.301)

**Table S2: Predicted reactivity of 3,7-dihydroxyphenoxazine**

| Compound | E _HOMO_ (a.u.) | E _LUMO_ (a.u.) | ƒ_H/L_ |
| --- | --- | --- | --- |
| 3,7-dihydroxyphenoxazine | -0.161 | -0.004 | 41.256 |
| AR | -0.017 | 0.104 | -0.167 |
| RFN | -0.218 | -0.106 | 2.055 |

E _HOMO_: Energy of the highest occupied molecular orbital

E _LUMO_: Energy of the lowest unoccupied molecular orbital

Although a precise prediction of the redox potential for the oxidation of 3,7-dihydroxyphenoxazine to RFN would require notably more complex calculations which go beyond the scope of the study, the reported quantum chemical descriptors reveal that 3,7-dihydroxyphenoxazine is markedly less stable than AR as encoded by the HOMO/LUMO fraction (the higher the ƒ_H/L_ ratio, the greater the predicted reactivity [[1]](#_ENREF_1)).

[1] Todeschini, R.C., V. (eds) Molecular Descriptors for Chemoinformatics: Volume I: Alphabetical Listing / Volume II: Appendices, References, Vol. 41. (Wiley-VCH Verlag GmbH & Co. KGaA, Weinheim, Germany; 2009).

**Supplemental Figures**

**
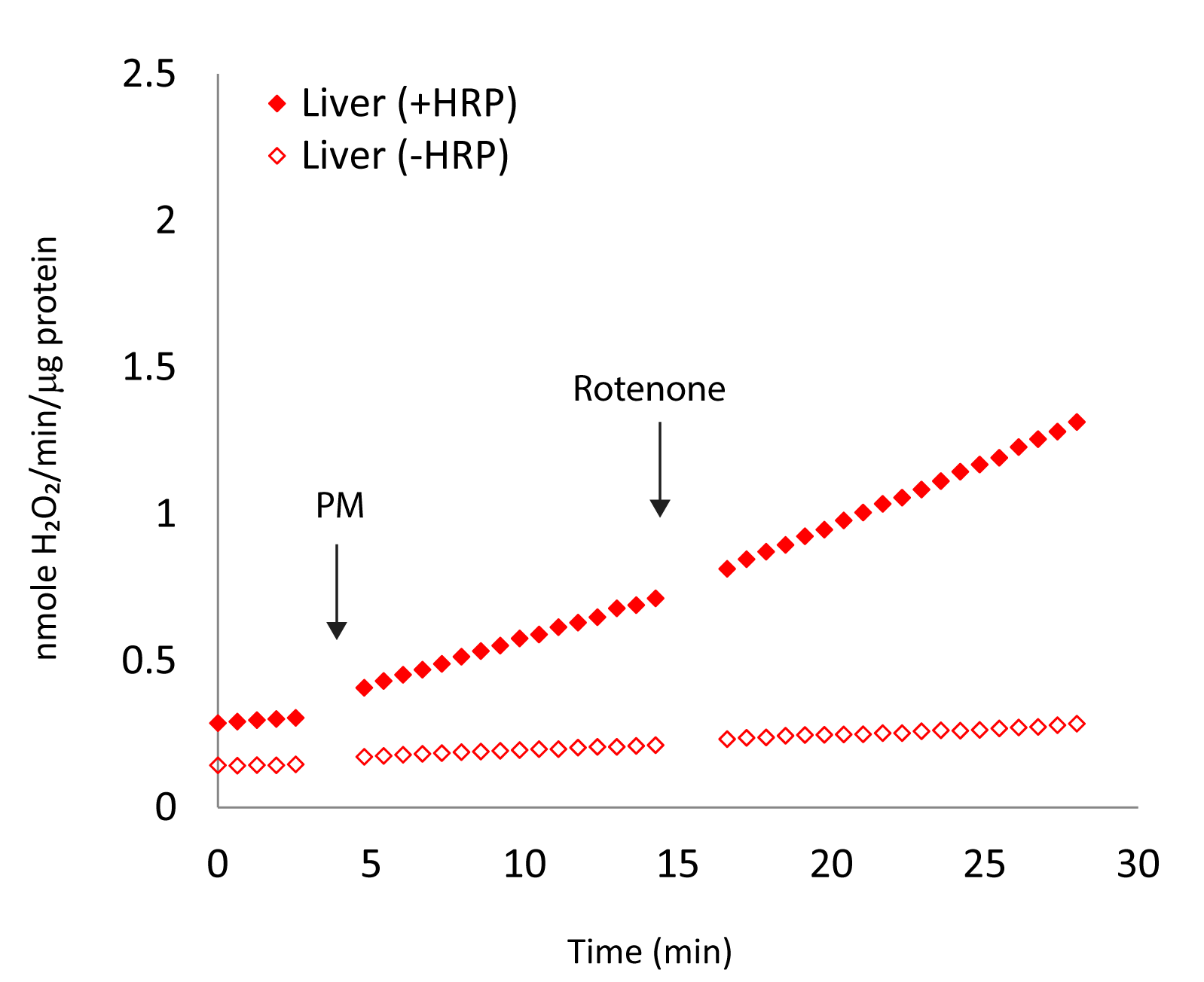
**

nmole H_2_O_2_/μg protein

**Supplemental Figure S1. H_2_O_2_ production by PMSF treated liver mitochondria** energized by 5 mM pyruvate and malate measured in the presence (filled symbols) and absence (open symbols) of HRP.

**
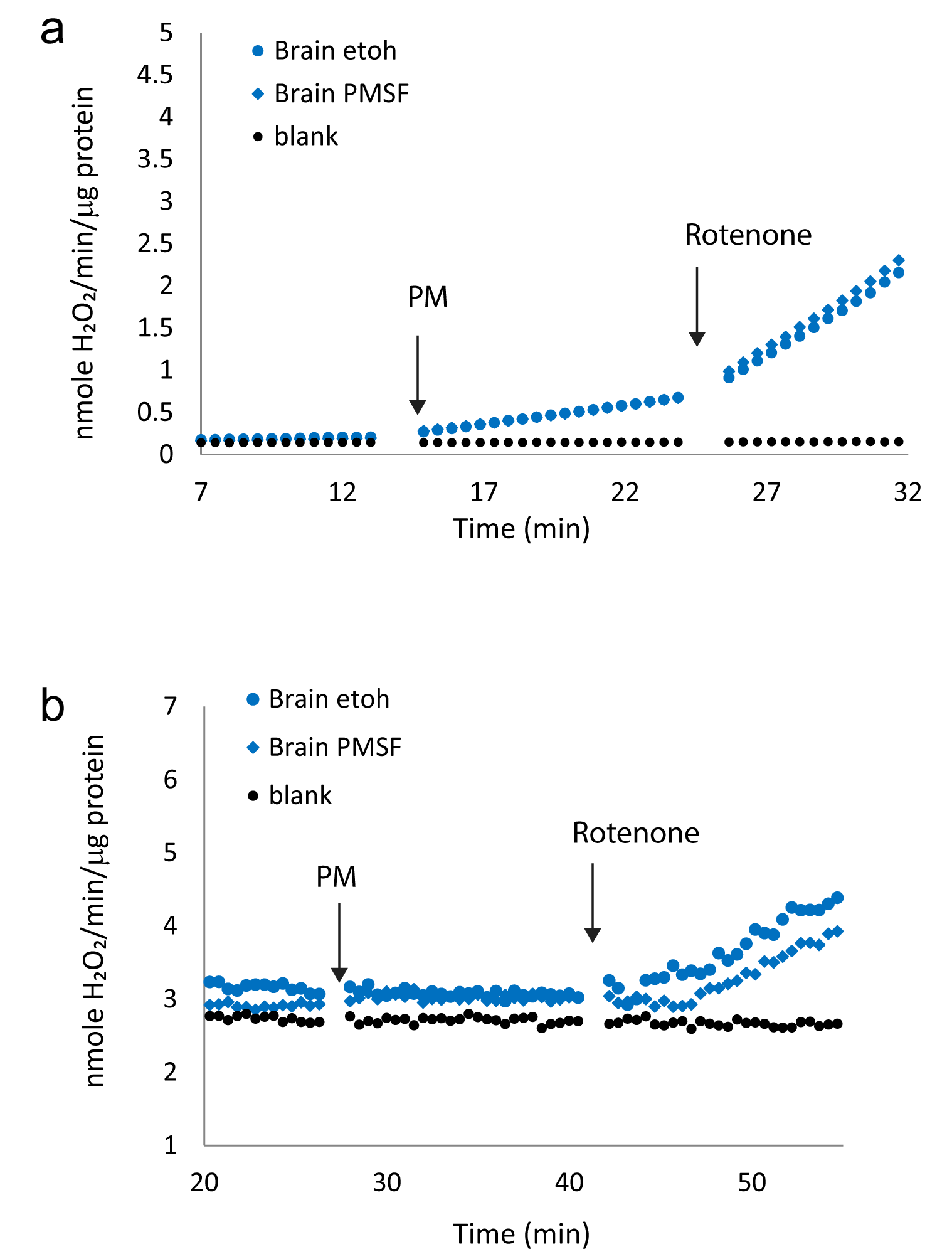
**

nmole H_2_O_2_/μg protein

nmole H_2_O_2_/μg protein

**Supplemental Figure S2.** **Representative traces from parallel experiments conducted with either AR plus PMSF (a) or Homovanillic acid (b) on purified brain mitochondria** with pyruvate and malate (5 mM) as substrate in the presence of HRP, followed by rotenone (2.5 µM). Symbols denote the following conditions: brain mitochondria in the presence (diamonds) or absence (circles) of 100 µM PMSF. Ethanol (etoh) was added as a control.

**
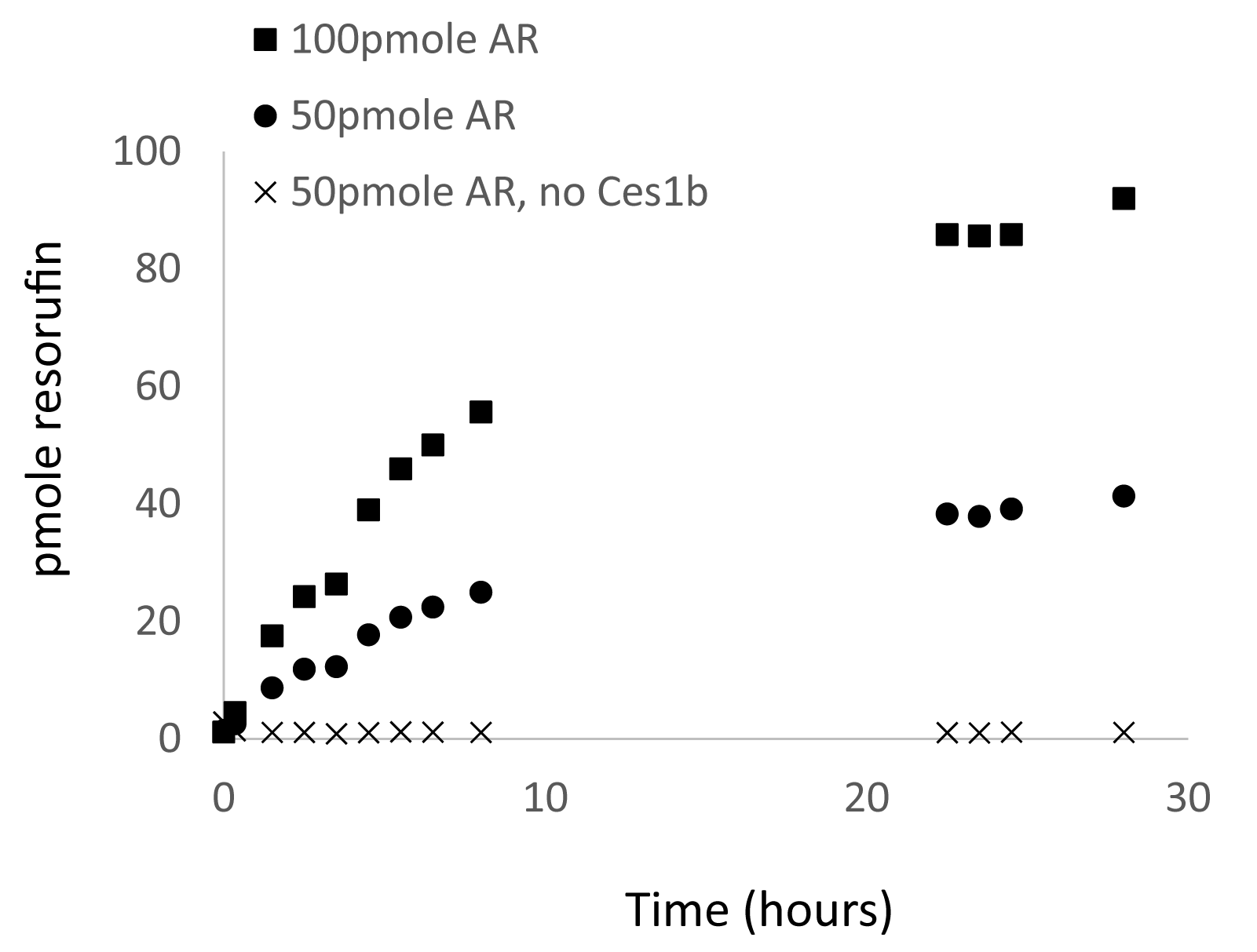
**

**Supplemental Figure S3.** **Resorufin appearance by the reaction of AR and CES1b (12.5 U/ml) with time.** The starting amounts of AR were either 100 pmole (squares) or 50 pmole (circles). After 28 hours, 92 % or 83 %, respectively, of AR molecules were converted to resorufin.


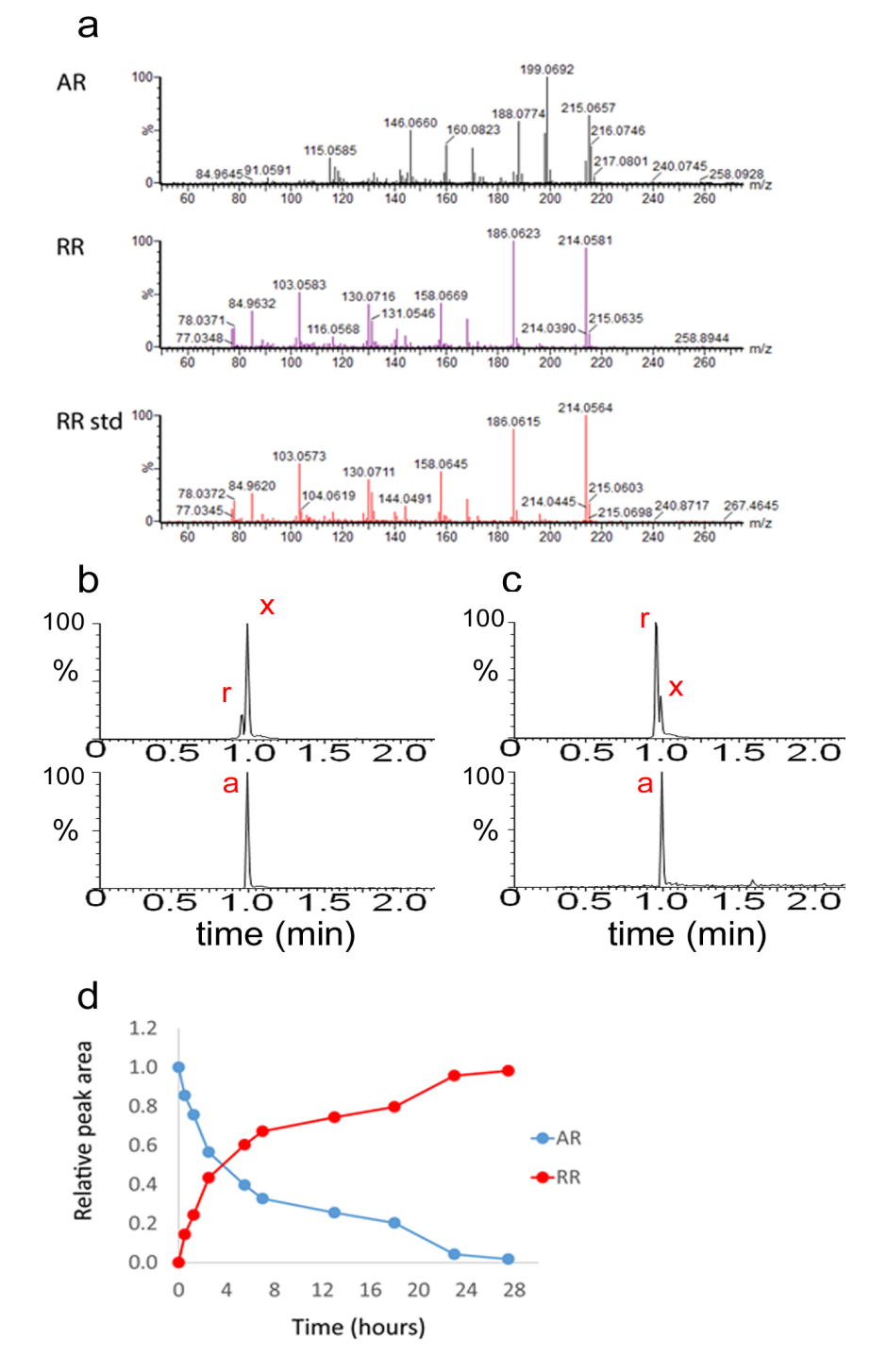


**Supplemental Figure S4.** **Conversion of AR to resorufin by CES1b.** **a)** MSMS spectra of an AR standard (panel AR), of the reaction product of the CES1b catalysed AR conversion eluting at 0.96 min (panel RR), and of a resorufin standard (panel RR std). **b) and c)** Extracted ion chromatograms of the reaction mix of the in vitro conversion of Amplex Red to resorufin (AR with 2.5U CES1b in 200 µl of H_2_O at 20 µM) after 30 min **(b)** and after 23 h **(c)**. The top chromatogram in both panels is the extracted ion chromatogram for m/z 214.050 (± 0.05 Th) and the bottom chromatogram is the extracted ion chromatogram for m/z 258.077 (± 0.05 Th). The peak annotated with ‘r’ corresponds to resorufin, the peak annotated with ‘x’ corresponds to an unknown contaminant in the reaction mixture that is present before the addition of CES, that does not change its relative intensity throughout the monitored reaction and that co-elutes with AR, but isobaric with resorufin and an MSMS spectrum that is very similar to that of resorufin. The peak labelled as ‘a’ corresponds to AR. **d)** Kinetics of the CES1b catalyzed production of resorufin from AR as monitored using LCMSMS. Time zero indicates product quantities before addition of CES1b.

**
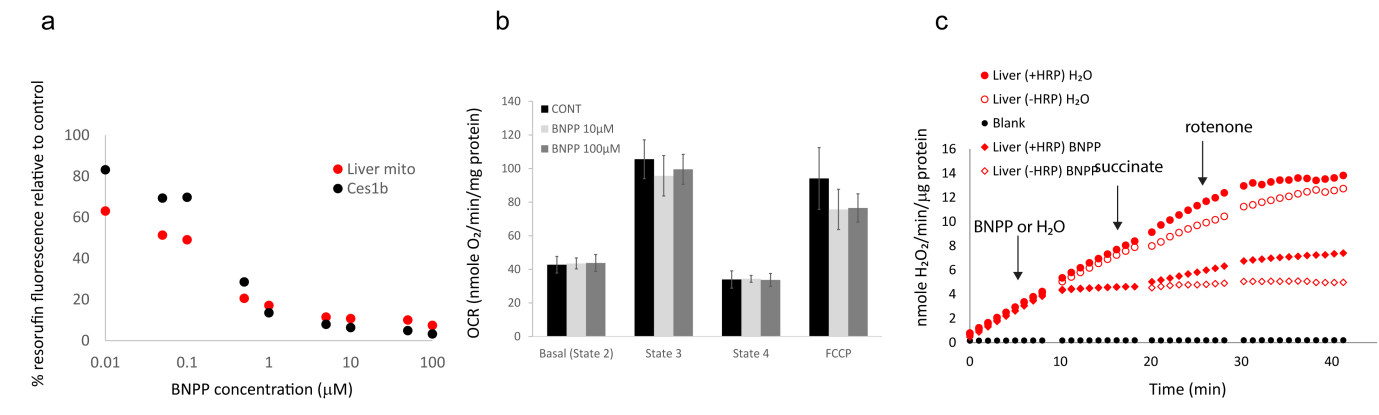
**

nmole H_2_O_2_/μg protein

**Supplemental Figure S5.** **Bis(4-nitrophenyl)phosphate (BNPP) inhibits the conversion of AR to resorufin by CES. a)** BNPP dose response curves for AR conversion to resorufin by Ces1b (Ces1b, black circles) or liver mitochondria (Liver mito, red circles). **b)** BNPP does not affect oxygen consumption rates (OCR) by liver mitochondria. OCR was measured in a Seahorse XF24 analyzer with 4 mM succinate as a substrate. Mitochondria were incubated without BNPP (CONT), or with 10 µM or 100 µM BNPP. OCRs for basal, state 3 (after ADP), state 4 (after oligomycin) and uncoupled state (after FCCP) are comparable under all conditions. **c)** BNPP blocks HRP-independent AR conversion. Representative kinetic traces from liver mitochondria-induced AR conversion to resorufin in the presence (diamonds) or absence (circles) of 10 µM BNPP. Water was added as a control (circles). Closed symbols indicate presence of HRP while open symbols indicate no HRP in the reaction mixture. Traces are shown in state 1 (no substrate), state 2 (succinate) and after blocking the electron flow at complex I by rotenone.


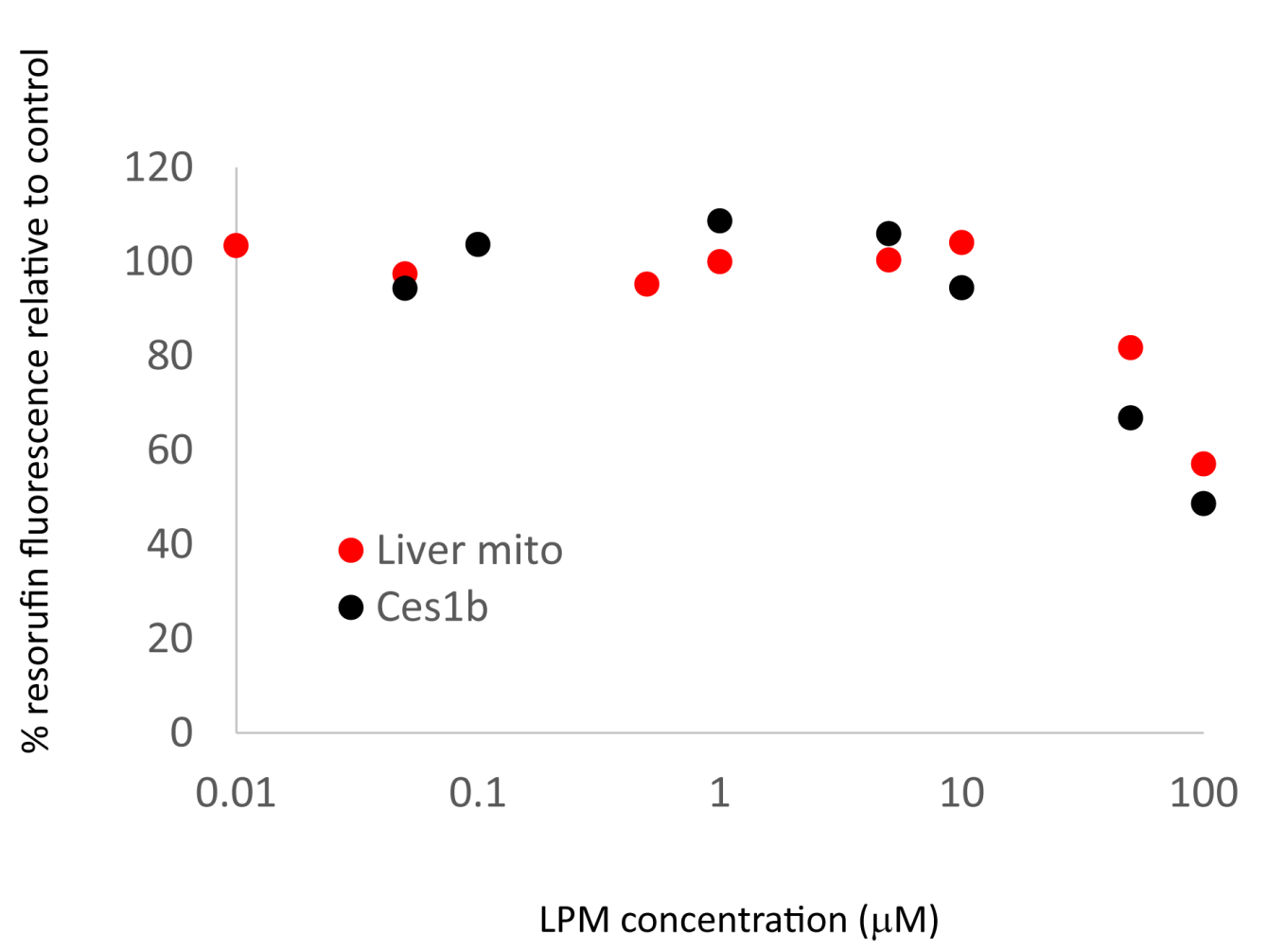


**Supplemental Figure S6.** **Loperamide (LPM) dose response curves for AR conversion by Ces1b** (Ces1b, black circles) **and liver mitochondria** (Liver mito, red circles)**.**


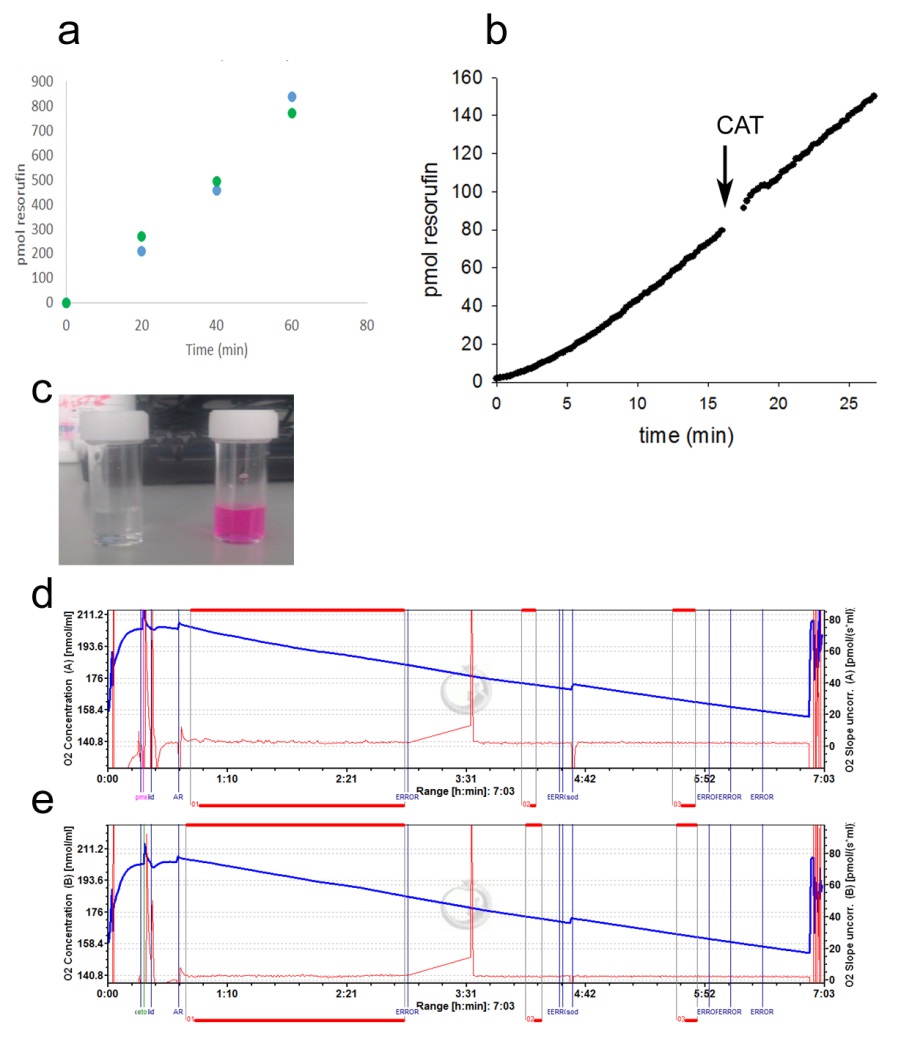


**Supplemental Figure S7. The conversion of AR to resorufin by Ces1b does not consume oxygen. a)** 50 μM AR were incubated with 25 U/ml CES1b under air (green) or under anaerobic conditions (blue) for the indicated times and the amount of resorufin produced was measured fluorimetrically. **b**) 50 μM AR were incubated with 10 U/ml CES1b for the indicated times and the amount of resorufin produced was measured fluorimetrically. 50 U/ml catalase (CAT) was added as indicated. **c)** 100 μM AR was incubated with 25 U/ml CES1b for 7 h at 37 °C in the dark with (left) or without (right) 100 μM PMSF while the reaction mixtures were subjected to oxygen consumption measurement by high-resolution respirometry in parallel. Both reactions contained 1 vol% ethanol (PMSF diluent). The photograph was taken immediately after the oxygen consumption measurement. **d)** and **e)** Oxygraph traces during the reaction of AR with CES1b in the presence (d) or absence (e) of PMSF. Oxygen consumption rates were calculated as 2.7 and 2.6 pmol O_2_/sec/ml, respectively, which is regarded as electrode drift.

**
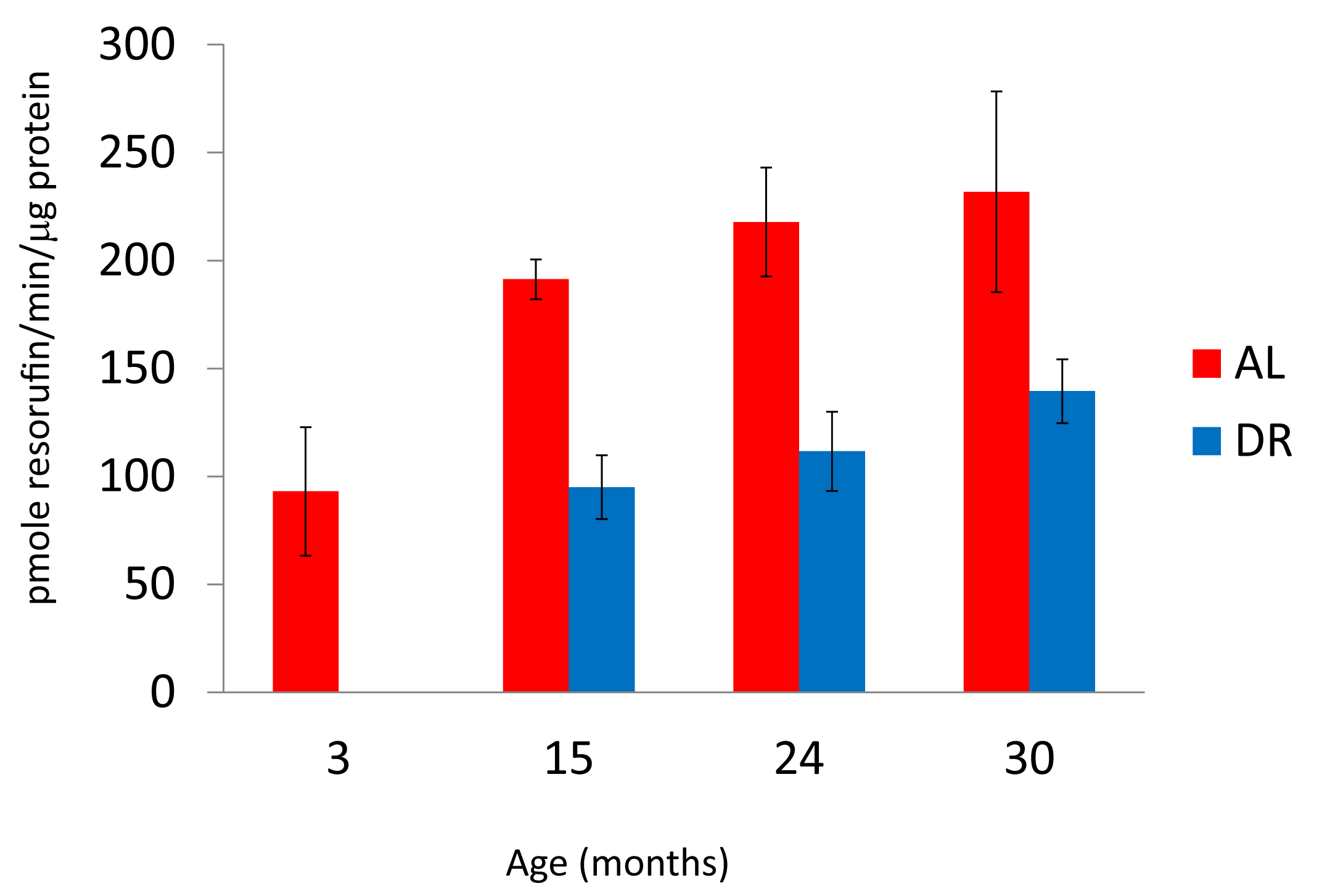
**

**Supplemental Figure S8. The rate of AR conversion to resorufin by mouse liver mitochondria at the indicated ages.** Mice were fed ad libitum (AL) or were dietary restricted (DR) to 60 % of AL food intake continuously from an age of 3 months. Data are mean ± SE from 4-5 animals per group. Effects of age and diet are significant with each p<0.001 (2-Way ANOVA).
